# Evaluation of a Rapid Lateral Flow Point-of-Care Test for Detection of *Cryptosporidium*

**DOI:** 10.4269/ajtmh.16-0132

**Published:** 2016-10-05

**Authors:** Molly E. Fleece, Jack Heptinstall, Shaila S. Khan, Mamum Kabir, Joel Herbein, Rashidul Haque, William A. Petri

**Affiliations:** 1 Department of Medicine, University of Virginia, Charlottesville, Virginia; 2 TechLab, Inc., Blacksburg, Virginia; 3 International Centre for Diarrhoeal Disease Research, Bangladesh (icddr,b), Dhaka, Bangladesh

## Abstract

A new rapid lateral flow fecal antigen detection test for *Cryptosporidium* was evaluated using diarrheal stool samples from a cohort of children in Bangladesh. The test had a sensitivity of 100% and a specificity of 94% when compared with enzyme-linked immunosorbent assay antigen detection.

Diarrheal diseases are a major cause of morbidity and mortality in the world.^1^,^2^ *Cryptosporidium* is an enteric protozoan parasite that is transmitted through the fecal-oral route, typically by consumption of contaminated food and water.^3^,^4^ The parasite has a low infectious dose resulting in diarrhea and abdominal pain.^4^,^5^ *Cryptosporidium* is a common cause of waterborne diarrheal disease worldwide, both in developing and developed countries as well as urban and rural areas; however, due to poor sanitation and urban crowding, *Cryptosporidium* infections are more prevalent in underdeveloped areas.^1^,^4^ Although infections do occur in immunocompetent hosts, immunocompromised hosts and children tend to have a more severe and prolonged disease course.^4^–^6^ There is need for a practical point-of-care diagnostic test that is rapid, reliable, and feasible for use in the field.

*Cryptosporidium* lateral flow (TechLab, Inc., Blacksburg, VA) is a newly developed immunochromatographic assay that qualitatively detects *Cryptosporidium* antigen in fecal specimens. It is a dipstick that uses a monoclonal antibody sandwich design to detect *Cryptosporidium* oocyst wall antigen. The assay flow begins with a diluted specimen that is drawn up via capillary action, the liquid fraction of which liberates membrane-embedded gold particles conjugated with anti-*Cryptosporidium* antigens. This mixture then flows to the visible reaction window where additional anti-*Cryptosporidium* antibodies are immobilized and capture antigen–gold complexes for a visual positive result.

The data presented here are of the first field test of the *Cryptosporidium* lateral flow focusing on the sensitivity and specificity of this rapid dipstick test. All diarrheal stool samples were collected from a cohort of children living in an urban slum in Bangladesh where *Cryptosporidium* is prevalent.^7^ The specimens were tested at the International Centre for Diarrhoeal Disease Research, Bangladesh. The samples were stored on average for 2 years at −20°C until testing in batches. Real-time polymerase chain reaction (PCR) testing had been performed on all diarrheal stool samples before this study.^8^ As a comparison of measurement of the presence/absence of *Cryptosporidium* antigen, enzyme-linked immunosorbent assays (ELISAs) were performed using the *Cryptosporidium II* test (TechLab, Inc.). The lateral flow was tested on 50 diarrheal stool samples known to be *Cryptosporidium* positive by PCR and 50 negative diarrheal stool samples. In addition, 100 randomly selected diarrheal stool specimens from children 6–12 months of age were tested using *Cryptosporidium* lateral flow and compared with the results of PCR testing.

Fecal samples were brought to room temperature and mixed thoroughly before beginning the test. Fifty microliters of specimen were transferred via pipette into the specimen dilution tube containing diluent (buffered protein solution). The sample end of a test strip was inserted into the specimen dilution tube. Results were read visually after 10 minutes. A sample was interpreted as positive if both test and control lines were present (Figure 1

**Figure 1.**
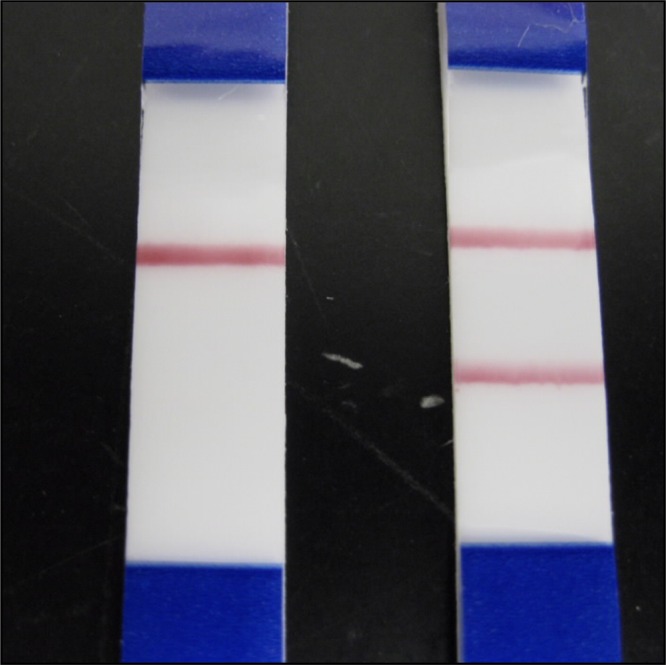
Lateral flow test for the detection of *Cryptosporidium* in stool specimens. The lateral flow on the left is an example of a negative test result where only the control line (upper) is positive. A positive test result is shown on the right with both the control and test lines visible.

). The color of the lines ranged from dark red to light pink, recognizing that color intensity did not correlate with strength of positivity. A sample was interpreted as negative if only the control line was visible. The test was considered invalid if the control line was absent.

We first tested 50 diarrheal stool samples known to contain *Cryptosporidium* DNA by PCR and 50 negative controls. Using ELISA as the reference standard for antigen detection, the *Cryptosporidium* lateral flow had a sensitivity of 100%, 94% specificity, 89% positive predictive value, and 100% negative predictive value (Table 1). Three of the four discrepant specimens (i.e., that were positive by *Cryptosporidium* lateral flow and negative by *Cryptosporidium II* test) were confirmed negative via PCR (with the fourth PCR positive).

We also evaluated the field adaptability of the lateral flow by testing 100 randomly selected diarrheal stool samples from the same cohort in Bangladesh, the vast majority of which did not have *Cryptosporidium*. There were no false positives: none of the 96 *Cryptosporidium*-negative samples had a positive lateral flow result. Of the four diarrhea samples with detectable *Cryptosporidium* DNA by PCR, the *Cryptosporidium* lateral flow detected one true positive sample with a C_t_ value 31.5. The three PCR (+) samples that were not detected by the lateral flow were most likely true negatives (i.e., *Cryptosporidium* was not the cause of diarrhea), as they had substantially lower amounts of *Cryptosporidium* DNA (C_t_ values of 35.2, 36.2, and 38.0). It has previously been shown that the strength of association of PCR (+) samples with diarrhea increases at higher pathogen loads.^9^

Available alternative rapid antigen detection dipstick tests include the Crypto Uni-Strip (Coris BioConcept, Gembloux, Belgium), RIDA QUICK Cryptosporidium (R-Biopharm, Darmstadt, Germany), and Crypto + Giardia dipstick (CLONIT, Milano, Italy).^10^–^14^ All these tests have comparable time to results and easy visual result interpretation; however, the other available rapid antigen detection tests above involve at least one additional step in comparison to the *Cryptosporidium* lateral flow test. We concluded that the *Cryptosporidium* lateral flow has a comparable sensitivity and specificity to the *Cryptosporidium II* ELISA and is rapid, reliable, and easy to use in the field.

## Figures and Tables

**Table 1:** Comparison of the *Cryptosporidium* lateral flow to the *Cryptosporidium II* ELISA for diarrheal stool samples

Assay type	*Cryptosporidium II* ELISA (+)	*Cryptosporidium II* ELISA (−)
Lateral flow (+)	34	4
Lateral flow (−)	0	62

ELISA = enzyme-linked immunosorbent assay.
